# Avian Influenza (H7N9) Virus Infection in Chinese Tourist in Malaysia, 2014

**DOI:** 10.3201/eid2101.141092

**Published:** 2015-01

**Authors:** Timothy William, Bharathan Thevarajah, Shiu Fee Lee, Maria Suleiman, Mohamad Saffree Jeffree, Jayaram Menon, Zainah Saat, Ravindran Thayan, Paul Anantharajah Tambyah, Tsin Wen Yeo

**Affiliations:** Queen Elizabeth Hospital, Kota Kinabalu, Malaysia (T. William, J. Menon);; KPJ Specialist Hospital, Kota Kinabalu (B. Thevarajah, S.F. Lee); Sabah State Health Department, Kota Kinabalu (M. Suleiman, M.S. Jeffree);; Institute of Medical Research, Kuala Lumpur, Malaysia (Z. Saat, R. Thayan);; National University of Singapore, Singapore (P.A. Tambyah);; Nanyang Technological University, Singapore (T.W. Yeo);; Tan Tock Seng Hospital, Singapore (T.W. Yeo);; Menzies School of Health Research, Darwin, Northern Territory, Australia (T.W. Yeo)

**Keywords:** H7N9, avian influenza, influenza, global travel, China, viruses, Malaysia

## Abstract

Of the ≈400 cases of avian influenza (H7N9) diagnosed in China since 2003, the only travel-related cases have been in Hong Kong and Taiwan. Detection of a case in a Chinese tourist in Sabah, Malaysia, highlights the ease with which emerging viral respiratory infections can travel globally.

Human infection with avian influenza (H7N9) virus was first reported from China in 2013 (*1*). Since then, ≈400 cases have been diagnosed in China and some in Hong Kong and Taiwan. Most patients were older adults with severe community-acquired pneumonia; risk for admission to an intensive care unit is 83%, and risk for death is 27%–36% (*2*,*3*). We report avian influenza (H7N9) virus infection outside greater China, in a Chinese tourist visiting Sabah, Malaysia.

## The Study

The patient was a 66-year-old woman from Guangzhou, Guangdong Province, China. She was not obese and had no relevant medical history. She worked on a household farm that cultivated vegetables but not animals. On January 30, 2014, she purchased from a poultry market 3 live chickens, which she slaughtered and cooked that day. According to the patient, these chickens and those at the market appeared healthy; she reported no contact with other birds. Two days later, she experienced cough, myalgia, and fever and consulted a local doctor who treated her symptomatically without performing laboratory or radiologic investigations. The woman subsequently went on holiday and flew to Peninsular Malaysia on February 4 and to Sabah, Malaysian Borneo, on February 6. She had persistent fever, worsening productive cough, arthralgia, abdominal pain, and diarrhea. On February 7, she sought care at Tuaran District Hospital for acute respiratory distress; she was intubated and transferred to a private specialist hospital in Kota Kinabalu, Sabah, Malaysia. At admission, she was placed in a negative-pressure isolation room with a portable high-efficiency particulate air filter that removes air particles >0.3 μm; staff observed airborne-transmission precautions.

Abnormal findings during initial examination were blood pressure 70/40 mm Hg, heart rate 96 beats/minute, and generalized crackles heard on lung auscultation. Hematologic, biochemical, and arterial blood gas results from samples obtained at admission (while the patient received 100% oxygen by mechanical ventilation) are summarized in the Table. Chest radiographs showed extensive dense pulmonary consolidations. The patient received dopamine, ceftriaxone, azithromycin, and oseltamivir. Culture of blood collected at admission grew methicillin-susceptible *Staphylococcus aureus*; tracheal aspirate results were negative, and cloxacillin was administered. The patient received ventilatory support with synchronized intermittent mandatory ventilation with a positive end-expiratory pressure of 10 cm H_2_O. On February 13 she was given methylprednisolone, which was discontinued on February 20 and replaced with oral prednisone on February 21. Ventilatory requirements gradually decreased, and she was extubated on February 22, after blood results normalized and oseltamivir was discontinued. However, on February 23, a low-grade fever and *Pseudomonas aeruginosa* bacteremia were found, and the patient was given meropenem and piperacillin-tazobactam. On February 28, her respiratory symptoms and fever recrudesced after discontinuation of oral prednisone. On the same day, oseltamivir and prednisone were given along with inhaled zanamivir, intravenous cefepime, and ciprofloxacin; her condition gradually improved. On March 7, oseltamivir and zanmivir were discontinued; on March 13, the patient was considered well and was discharged with a tapering dose of prednisone. She returned to China on March 16. A time line of her travel and hospital course is detailed in the Figure.

**Table:** Hematologic and biochemical values for Chinese patient with avian influenza (H7N9) virus infection at hospital admission, Malaysia, February 7, 2014

Laboratory test	Value (reference range)
Blood counts	
Hemoglobin, g/dL	12.7 (12.0–18.0)
Leukocyte, cells/mm^3^	10.2 × 10^3 ^(3.5–12 × 10^3^)
Neutrophils, cells/mm^3^	9.2 × 10^3 ^(2.5–7.5 × 10^3^)
Lymphocytes, cells/ mm^3^	276 (1.0–4.8 × 10^3^)
Platelets, cells/mm^3^	150 × 10^3 ^(150–400 × 10^3^)
Serum tests	
Creatinine, mmol/L	87 (50–110)
Sodium, mmol/L	140 (135–145)
Potassium, mmol/L	3.6 (3.5–5.1)
Chloride, mmol/L	102 (96–106)
Total protein, g/L	57 (60–80)
Albumin, g/L	23 (35–50)
Globulin, g/L	34 (25–40)
Total bilirubin, μmol/L	6.6 (2–28)
Alanine aminotransferase, IU/L	92 (7–40)
Aspartate aminotransferase, IU/L	208 (5–35)
C-reactive protein, mg/L	263.76 (<3)
Arterial blood measurements*	
pH	7.41 (7.35–7.45)
Partial pressure of oxygen, mm Hg	65 (75–100)
Partial pressure of carbon dioxide, mm Hg	4.3 (38–42)
Lactate, mmol/L	1.4 (0–2)

*Obtained while patient was receiving 100% oxygen.

**Figure:**
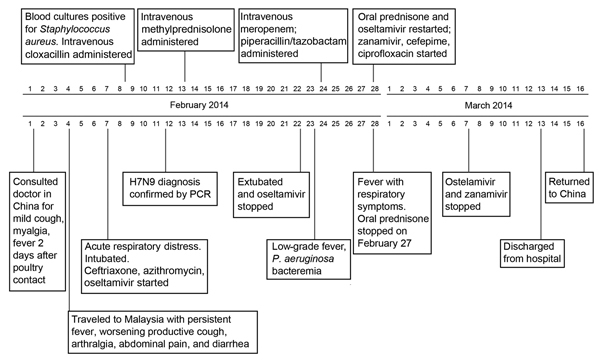
Time line of the travel dates and hospital course of avian influenza (H7N9) virus infection in Chinese tourist to Malaysia.

According to Malaysian Ministry of Health criteria, infection with influenza (H7N9) virus is suspected in persons with severe respiratory illness and a history of travel from affected areas in China. For this patient, on February 7, clinicians from district and specialist hospitals sent throat swabs in viral transport media (Copan Diagnostics, Inc., Murrieta, CA, USA) to the National Influenza Centre at the Institute of Medical Research in Kuala Lumpur for virus studies. Real-time reverse transcription PCR was used to test for the following viruses, as described (*4*): influenza A, influenza B, influenza A(H1N1) pdm09, influenza H1 seasonal, influenza H3 seasonal (all by using Centers for Disease Control and Prevention protocols [*5*]), influenza H7 avian (Centers for Disease Control and Prevention, Chinese National Influenza Centre [*1*], and local protocols), and influenza N9 (Chinese National Influenza Centre protocol). Results were positive for influenza A and influenza H7 and N9: results remained positive on repeated swab samples collected on February 13 and 22. Sequencing of the hemagglutinin and neuraminidase genes revealed that these strains were closely related to strains from Guangzhou and Guandong (*4*) but did not reveal the mutation associated with oseltamivir resistance: R294K (N9 numbering) (*6*). No virus was detected in samples collected on February 26 and 28. 

During February 7–16, officials from the Malaysian Public Health Department and Sabah State Health Department identified 191 persons who had had contact with the patient; 6 were symptomatic, but nasopharyngeal swab specimens were negative for influenza. The symptomatic contacts included tour group members and personnel from various hotels, restaurants, airlines, tourist destinations, airports, and hospitals.

## Conclusions

The rapid diagnosis of avian influenza (H7N9) virus infection outside China and Taiwan in a Chinese tourist traveling to Sabah, Malaysia, highlights the value of a high index of suspicion by medical staff, awareness and adherence to national guidelines, and good laboratory services (*4*). The clinical features of the patient were similar to those previously reported from China: median age of affected persons was 61 years, and >80% of patients reported exposure to live poultry and experienced pneumonia or respiratory failure (*7*). Virus was detected by reverse transcription PCR after the patient had received oseltamivir for 2 weeks, as has been reported (*6*), although the significance of prolonged detection is unclear because the patient’s clinical condition improved markedly, no oseltamivir resistance mutations were found (*4*), and virus persistence may have be associated with steroid use. The patient’s condition deteriorated after taking oseltamivir 1 week after symptom onset and ventilatory requirements increased, possibly because of secondary *S. aureus* infection, a well-known complication of influenza (*8*). The patient was given methylprednisolone after a week of hospitalization; although she subsequently improved, it was unclear if this was in response to the antimicrobial drugs, the corticosteroids, or the natural course of the infection. Anecdotal reports describe clinical improvement of a patient in Taiwan with influenza (H7N9) pneumonia after receipt of corticosteroids (*9*), but such improvement has not been supported by larger studies of influenza (H7N9) patients (*3*) or studies of adults in Vietnam infected with avian influenza (H5N1) virus (*10*,*11*).

Of the ≈400 cases of human avian influenza (H7N9) infection diagnosed in China since 2003, the only travel-related cases were in Hong Kong and Taiwan, which have close geographic, economic, and cultural ties to China and extensive bidirectional travel. This case highlights the ease with which emerging viral infections can travel globally. On a map of recent air travel from China, major destinations identified (*12*) were Taiwan, Hong Kong, Malaysia, and Singapore; dozens of flights went to major cities in Europe and North America, which received hundreds of visitors directly from China weekly. In the first 3 quarters of 2013, an estimated 72.5 million tourists left China (*13*). To ensure accurate identification and appropriate management of emerging novel respiratory viral infections, clinicians in destination countries need to obtain detailed travel histories from tourists and returning travelers 

The influenza (H7N9) virus is not easily transmissible among humans, and our investigations did not find any evidence of spread to the patient’s fellow travelers, medical staff, or other contacts. However, the virus has the potential to adapt to mammalian hosts over time (*14*,*15*). Clinicians and public health authorities need to be alert to the latest epidemiologic information on emerging respiratory viruses; local capacity to isolate, diagnose, and treat illness in travelers with unusual respiratory viral infections is also needed.
